# The use of whole-body cryotherapy: time- and dose-response investigation on circulating blood catecholamines and heart rate variability

**DOI:** 10.1007/s00421-020-04406-5

**Published:** 2020-05-30

**Authors:** Julien Louis, Dimitri Theurot, Jean-Robert Filliard, Marielle Volondat, Benoit Dugué, Olivier Dupuy

**Affiliations:** 1 Research Institute for Sport and Exercise Sciences (RISES), Liverpool John Moores University, Byrom Street, Liverpool, L3 3AF UK; 2 Laboratoire MOVE (EA 6314), Faculté des Sciences du Sport, Université de Poitiers, Poitiers, France; 3 Medical Department, French Institute of Sport (INSEP), 11 avenue du tremblay, 75012 Paris, France

**Keywords:** Cryostimulation, Autonomic nervous system, Body temperature

## Abstract

**Purpose:**

A predominance of parasympathetic drive is observed following cold exposure. Such modulation of the autonomic nervous system (ANS) is associated with faster post-exercise recovery. Within this context, whole-body cryotherapy (WBC) has been spreading in sport medicine, though the optimal temperature and frequency are unclear. The aim of this study was to examine the effects of different cryotherapy conditions on the sympathovagal balance.

**Methods:**

Forty healthy males were randomly assigned into five different groups (− 110 °C, − 60 °C, − 10 °C, control temperature [≃ 24 °C]) and undertook 5 WBC sessions over 5 consecutive days. Cardiac autonomic activity was assessed through heart rate variability (HRV) using power density of high frequency (HF), root-mean square difference of successive R–R intervals (RMSSD) and sympathovagal balance (LF/HF). Systemic sympathetic activity was assessed via circulating blood catecholamines.

**Results:**

Mean weekly RMSSD (pre: 48 ± 22 ms, post: 68 ± 29 ms) and HF (pre: 607 ± 692 ms^2^, post: 1271 ± 1180 ms^2^) increased (*p *< 0.05) from pre to post WBC, only in the − 110 °C condition. A rise in plasma norepinephrine was found after the first − 110 °C WBC session only (pre: 173 ± 98, post: 352 ± 231 ng L^−1^, *p *< 0.01); whereas, it was not significant after the 5th session (pre: 161 ± 120, post: 293 ± 245 ng L^−1^, *p *= 0.15).

**Conclusion:**

These results suggest that one − 110 °C WBC exposure is required to stimulate the ANS. After five daily exposures, a lower autonomic response was recorded compared to day one, therefore suggesting the development of physiological habituation to WBC.

## Introduction

Cryotherapy consists in exposing the body to cold temperature. It is traditionally implemented in the form of ice packs applied onto the body or cold-water immersion where part or the entire body is immersed (Dugué and Leppanen 2000). Whole-body and partial-body cold air cryostimulation (WBC and PBC) are now presented as modern cryotherapy techniques with an ever-growing popularity in the sport and medical sphere (Bleakley et al. 2014). The use of WBC is multiple, including post-exercise recovery for athletes (Dupuy et al. 2018), pain soothing in inflammatory conditions (Banfi et al. 2009; Guillot et al. 2014; Dupuy et al. 2018) as well as well-being and sleep management (Bouzigon et al. 2014; Douzi et al. 2019a, b). The physiological and psychological actions of WBC originate from a reduction in skin temperature which triggers a cascade of reactions starting with a peripheral vasoconstriction through sympathetic tuning (Westerlund et al. 2003). As a response to vasoconstriction, peripheral blood volume is redirected to the core of the body, leading to an increase in pre-load and central blood pressure (Westerlund et al. 2004a, b). Such reaction stimulates baroreceptors and leads to a diminution of the sympathetic nerve activity and an increase in vagal control of the myocardium (Zalewski et al. 2013). As such, it is well established that WBC is effective in increasing post-exercise and resting heart rate variability (HRV), an indicator of increased parasympathetic tone activation (Westerlund et al. 2006; Schaal et al. 2013; Hausswirth et al. 2013; Zalewski et al. 2014).

Many variables are susceptible to affect the magnitude of the physiological responses to WBC. Among them, the exposure duration, temperature and the sessions’ frequency may be the most influential. For instance, Louis et al. (2015) demonstrated that with an identical decrease in skin temperature, head exposure to cold during a WBC session led to higher activity of vagal-related HRV indices. These results were similar to previous research which revealed a larger parasympathetic stimulation after WBC compared to PBC (Hausswirth et al. 2013). Selfe et al. (2014) tested the effects of three WBC exposure durations (1, 2 and 3 min) on skin temperature and hemodynamic responses, and found that at least 2 min of exposure was necessary to significantly reduce skin temperature and trigger physiological responses. Similarly, Fonda et al. (2014) compared four different exposure durations (1 min 30 s, 2 min, 2 min 30 s and 3 min) to PBC and stated that at least 2 min 30 s of exposure was required to significantly reduce skin temperature. In parallel, the influence of frequency and temperature of exposure remains to be elucidated. In one of the first chronic WBC protocols, Westerlund et al. (2004a) reported a similar increase in blood pressure following a unique WBC exposure compared to repeated exposures (3 times a week for 3 months). Similarly, Lubkowska and Szyguła. (2010) later reported no difference in blood pressure after 1 and 15 WBC exposures. Interestingly, albeit not using WBC, Mäkinen et al. (2008) recorded a decrease in sympathetic activity concomitant to a minor increase in parasympathetic activity after acute cold exposure (2-h whole-body cold-air exposure to 10 °C) and these effects were accentuated after habituation to cold (protocol repeated once daily for 10 consecutive days). Even though the cold stimulation was not as high as with WBC, these results reinforce the fact that the frequency and temperature of cold exposure play a major role in WBC.

It is clear that the characteristics of each WBC session, namely the temperature of the cryotherapy chamber, the duration and the frequency of exposure must be controlled and adapted to maximize its benefits. A better understanding of how these characteristics influence human physiological functions is required and warrants more research. Ultimately, bespoke WBC protocols could be proposed. Accordingly, the aim of this study was to investigate the acute and repeated effects of three different WBC exposure temperatures on skin temperature and on the subsequent modulation of the autonomic nervous system in healthy males.

## Materials and methods

### Participants

Forty healthy males volunteered to participate in this study (see Table 1 for characteristics). Before the experiment, a physician examined all the participants to check they did not present contraindications to cold exposure such as cold hypersensitivity (Raynaud’s syndrome), heart condition or circulatory pathology. All the participants were recreational athletes, between 20 and 55 years old, and were not accustomed to cryotherapy exposures. Since body mass can influence the physiological response to cryostimulation, body composition was controlled and measured using an 8-point bio-impedance device (InBody 720; 1–1000 kHz, Biospace company, Ltd., Seoul, Korea) validated for accuracy and repeatability. All the participants were requested not to smoke, nor drink alcohol or hot drinks within the 4 h prior to each cold exposure. In addition, participants were required to restrain their physical activity for 24 h prior to each laboratory session. All participants were volunteers and were informed about the study protocol, and their rights according to the Declaration of Helsinki. Participants gave their written informed consent and the study was approved by the local Ethics Committee (Ile-de-France, Paris).

**Table 1:** Characteristics of participants composing the four experimental groups (Control, − 10 °C, − 60°C, − 110 °C)

	Control	− 10 °C	− 60 °C	− 110 °C
N	10	10	10	10
Age (year)	33.9 ± 12.3	33.3 ± 11.0	34.8 ± 9.1	34.4 ± 13.8
Height (m)	1.77 ± 0.06	1.80 ± 0.07	1.74 ± 0.09	1.77 ± 0.05
Body mass (kg)	74.4 ± 11.8	82.5 ± 17.3	69.9 ± 12.3	79.2 ± 10.6
BMI (kg m^−2^)	23.6 ± 3.1	25.5 ± 4.1	23.0 ± 2.2	25.2 ± 10.6
Fat mass (%)	13.3 ± 6.0	16.5 ± 6.4	14.2 ± 4.1	15.1 ± 5.4

Data are presented as mean ± SD; BMI: Body Mass Index

### Study design

**Fig. 1:**
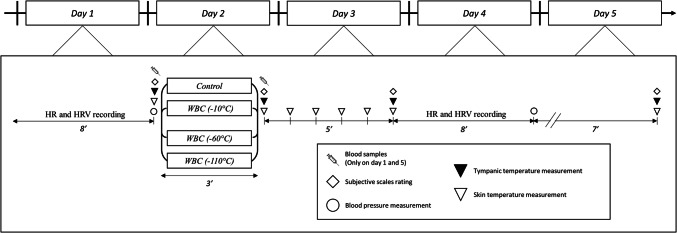
Study design. *HR* heart rate, *HRV* heart rate variability, *WBC* whole-body cryotherapy

This study was conducted to understand the effects of different temperatures of WBC on the autonomic nervous system and, thus, test a potential time- and dose-response effect of WBC. Three different temperatures of WBC were compared in this study (− 10 °C, − 60 °C, − 110 °C) and also compared with a control trial (ambient temperature, 24 °C). The main purpose was to determine the optimal temperature of WBC, i.e. inducing the greatest stimulation of the parasympathetic activity, and thus the greatest benefits for users. In separate weeks, 4 groups of 10 participants were exposed either to five (one session per day) − 10 °C, − 60 °C, or − 110 °C WBC sessions or to a control trial (24 °C) for 3 min. On each testing day, physiological measurements were performed immediately before and within the 20 min following the exposure (Fig. 1).

### Cryotherapy sessions

WBC sessions were administered in a specially built, temperature-controlled unit (Zimmer Elektromedizin, GmbH, Ulm, Germany), which consisted of three rooms (− 10 °C, − 60 °C and − 110 °C). For all WBC sessions, depending on the treatment group, participants stayed in the − 10 °C room, or −  60 °C, or passed through the warmer rooms and remained in the − 110 °C room for 3 min. The entire cooling process was automatically controlled and the temperatures of all rooms remained constant throughout the experiment. A familiarisation session was previously organised with a time exposure reduced to 1 min, and cryostimulation sessions were administered under medical supervision. Prior to each WBC session, the participants were instructed to towel-dry potential sweat, wear a bathing suit, surgical mask, earband, triple-layer gloves, dry socks, and sabots. All jewellery, piercings, glasses and contact lenses were removed before entering the WBC rooms. During the 3-min WBC session, to avoid muscle tension, the machine operator instructed the participants to walk slowly in the chamber and to lightly flex and extend their elbow.

### Measurements

#### Skin and tympanic temperatures

Skin temperature was assessed by a ThermoVision SC640 Thermal imaging camera (Flir Systems, Danderyd, Sweden) in accordance with the standard protocol of infrared imaging in medicine (Ring and Ammer 2012). The camera, with the emissivity set in the range of 0.97–0.98, was connected to a personal computer with dedicated software (Thermacam Researcher Pro 2.10, Flir systems, Danderyd, Sweden). The camera was mounted on a tripod and positioned in a way to focus on the entire body. The distance between the camera and the subject ranged from 2.5 to 3.0 m (depending on the height and the size of the participant). Immediately after the WBC session, participants were instructed to remain standing for 5 min in a temperate room. The thermograms of the chosen body regions of interests were performed before (Pre) and during the first 5 min after the WBC session (post–P5) and 20 min after (P20), in the temperate room where the temperature was stabilised (24 °C). Participants were also asked to turn around immediately after the exposure to cold (Post), and at the end of each minute (P1–P5) to perform thermograms of the back of the body. Finally, a last front and back thermogram was performed 20 min after the end (P20) of the WBC sessions. Twenty body regions of interest were chosen to study as thoroughly as possible the evolution of skin temperature before and after the cold exposure. Ten regions corresponded to the front face of the body (i.e. torso, abdominal, right and left forearms, right and left arms, right and left thighs, and right and left legs) and 10 regions corresponded to the back face (i.e. upper back, lower back, right and left forearms, right and left arms, right and left thighs, and right and left legs) covering almost the whole body. For more clarity in the presentation of the results, a mean temperature for the whole body was calculated by averaging the skin temperature recorded for the 20 regions of interest.

As an additional measurement technique before and after the WBC, an estimation of the core temperature (tympanic temperature, Ttymp) was performed by a tympanic thermometer (Braun Thermoscan^®^ Pro 4000, NY, USA). This measurement was performed at Pre, Post, P5, and P20.

#### Blood pressure, heart rate, and HRV indices of parasympathetic activity

For five consecutive days, heart rate (HR) was recorded at pre and P5. For each measurement condition, participants were comfortably positioned in a supine position on a medical bed for 8 min. This test was conducted in a dark and quiet room, to avoid HR fluctuations. Additionally, the participants were asked to remain still and not to talk. For all resting HR recordings, *R*–*R* intervals were recorded continuously with a Suunto MemoryBelt HR monitor with a sampling rate of 1000 Hz (MemoryBelt, Suunto Oy^®^, Vantaa, Finland).

*R*–*R* interval data files were transferred to the computer using Suntoo Training Manager Software and were further analysed using specialised heart rate variability (HRV) analysis software (Nevrokard^®^ aHRV, Izola, Slovenia). An experienced investigator visually identified and manually removed any occasional ectopic beats and artefacts. Power spectral density analysis was performed using a fast Fourier transform with a non-parametric algorithm. The total power density was divided into two spectral components. These components are integrals from the low-frequency (0.04–0.15 Hz) and high-frequency (0.15–0.50 Hz) spectral bands. The distribution of the power and the central frequency of low- and high-frequency bands are not fixed but may vary in relation to changes in autonomic modulations of the heart period. HRV in the high-frequency range is so rapid that it may only be mediated by the parasympathetic nervous system. However, part of the high-frequency power seems to be caused by respiration-induced changes in the intrathoracic pressure and blood volume. In the low-frequency range, both the sympathetic and the parasympathetic nervous system can affect HRV. Our analysis was, therefore, restricted to HRV indicators of parasympathetic modulation, namely the power density of high frequency (HF), the root-mean square difference of successive normal R–R intervals (RMSSD), a time-varying index, and the LF/HF ratio. Mean HR was also analysed. All HRV indices of parasympathetic activity were calculated from the last 4 min of the 8-min HR recordings (Le Meur et al. 2013; Bourdillon et al. 2017). Participants were allowed to breathe spontaneously during the measurements (Larsen et al. 2010), but for all HRV samples, it was verified that the respiration rate always remained in the high-frequency range (HF, 0.15–0.50 Hz). When this assumption was not met, the test was not retained for subsequent analysis.

Systolic and diastolic blood pressures (Sys BP and Dia BP) were also recorded at the end of the 8-min resting period, using an oscillometric sphygmomanometer (705 IT, Omron, Kyoto, Japan) positioned on the left arm, while the person was still in a lying position.

#### Blood analyses

On two occasions (before and after the 1st and 5th WBC session), blood samples were collected from a superficial forearm vein using standard venepuncture technique. For each blood sampling, 33 ml was directly collected into EDTA tubes (Greiner Bio-one; Frickenhausen, Germany). Blood samples were immediately centrifuged at 3000 rev min^−1^ for 10 min at +4 °C to separate plasma from red blood cells. The obtained plasma sample was then stored in multiple aliquots (Eppendorf type, 1500 μL per samples) at − 80 °C until analysis. From these samples, epinephrine and norepinephrine were determined in plasma by enzyme-linked immunosorbent assay with commercially available high sensitivity ELISA kits (Alpco Dagnostics, Salem, USA). All blood samples were analysed in duplicate at specific wavelength (450 nm) on a spectrophotometer Dynex MRXe (Magellan Biosciences, Chelmsford, MA, USA). To avoid inter-assay variation, all blood samples were analysed in a single batch at the end of the study. Intra-assay coefficients of variation were 6.8% and 9.7% for epinephrine and norepinephrine, respectively. Analytical sensitivity was 10 and 36 ng L^−1^ for epinephrine and norepinephrine, respectively.

#### Thermal and comfort sensations

Thermal and comfort sensations of participants were recorded at Pre, Post, P5 and P20 using thermal and comfort scales of perception (Smolander et al. 2004). The participants rated their thermal sensation with a nine-point standard scale before and after WBC sessions by answering the question ‘How are you feeling now?’. They were instructed to answer by instinctively giving a number ranged from 4 to − 4 (4 = very hot, 3 = hot, 2 = warm, 1 = slightly warm, 0 = neutral, − 1 = slightly cool, − 2 = cool, − 3 = cold, − 4 = very cold). Thermal comfort was also rated with a five-point scale by answering the question ‘How do you find this?’. Participants were instructed to answer by instinctively giving a number ranged from 0 to 4 (0 = comfortable, 1 = slightly uncomfortable, 2 = uncomfortable, 3 = very uncomfortable, 4 = extremely uncomfortable).

### Statistical analysis

All data are expressed as mean ± standard deviation (SD). A three-way analysis of variance (Condition [Temperature] × Day × Time [Pre–post]) for repeated measures was performed to analyse the effects of the different modalities of cryotherapy (− 10 °C, − 60 °C, − 110 °C, or no cryostimulation) over time, with catecholamines concentrations, HRV parameters, BP, skin temperature, tympanic temperature, and perceived sensations as dependent variables. The Newman–Keuls post hoc test was used to determine the between-means differences if the analysis of variance revealed a significant interaction between conditions, time, and/or day. For all statistical analyses, a *p* < 0.05 value was accepted as the level of significance. Threshold values for Hedge’s effect size (ES) interpretation were ≤ 0.2 (trivial), > 0.2 (small), > 0.5 (moderate), and > 0.8 (large).

## Results

### Skin and tympanic temperatures

**Fig. 2:**
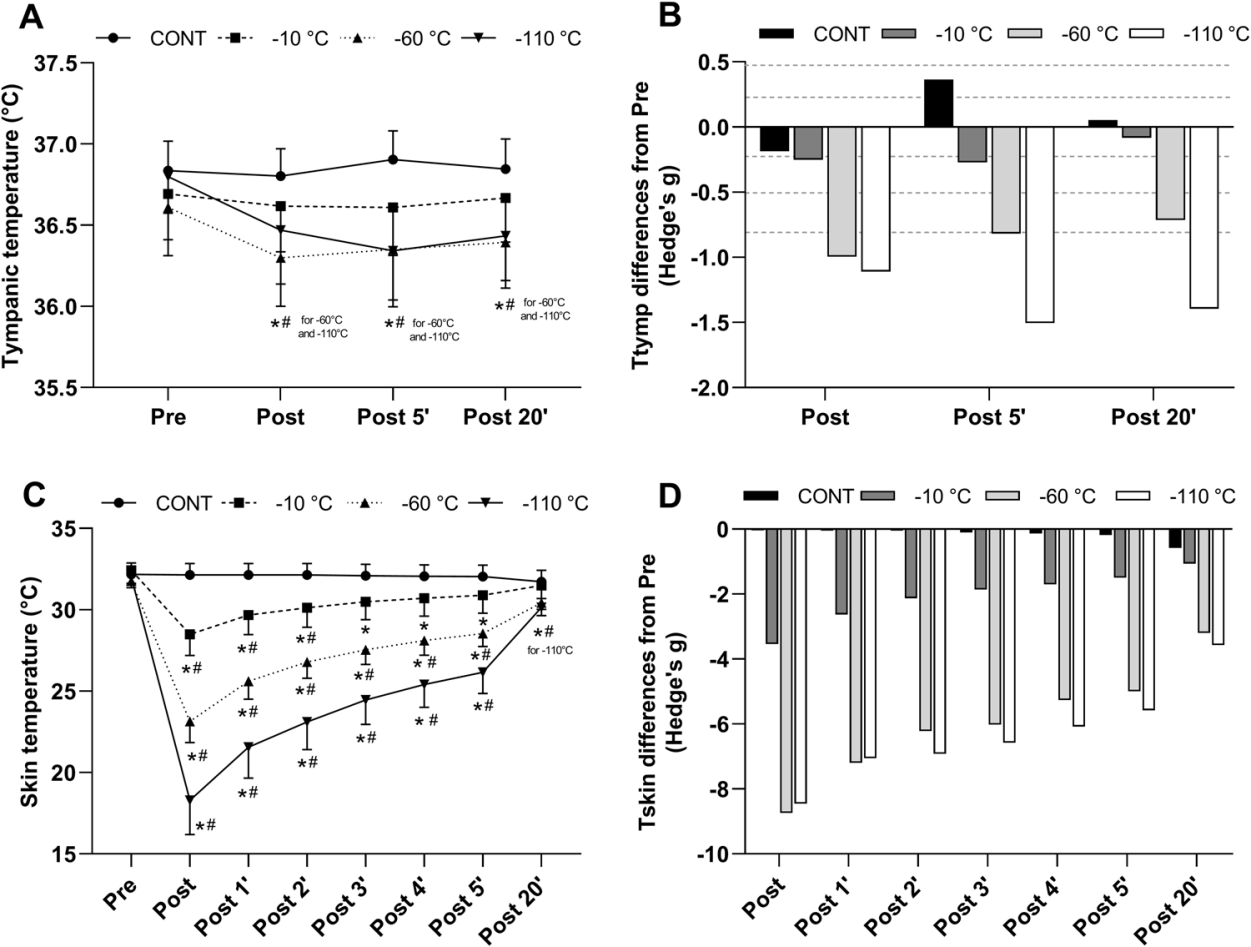
Mean and SD values of tympanic (**a**) and skin temperature (**c**) of the whole body before (Pre), immediately after (Post) and for 20 min (P1–P20) after a whole-body cryotherapy (WBC) session at − 10 °C, − 60 °C, − 110 °C, and in the control condition (CONT). Values are means obtained for 5 WBC sessions. *Significantly (*p *< 0.05) different from Pre;# Significantly (*p *< 0.05) different from CONT. The magnitude of change (Hedge’s g) from pre for tympanic (**b**) and skin temperature (**d**). Horizontal dashed lines correspond to very small (< 0.2), small (0.2 < ES < 0.5), moderate (0.5 < ES < 0.8), large (0.8 < ES < 1.2) effects

The evolution of Tskin and Ttymp for the different WBC conditions is presented in Fig. 2. Baseline Tskin of the whole body was similar between all conditions and no differences were found between days of exposure. The ANOVA revealed a main effect of time and condition (*p* < 0.005). From post to P2, for all WBC conditions Tskin was lower than the control condition. Tskin was lower in the − 60 °C condition compared to − 10 °C and lower in the − 110 °C compared to all the other conditions. From P3 to P5, Tskin in the − 60 °C and the − 110 °C conditions was lower than − 10 °C and the CONT condition; while a difference was also recorded between − 60 °C and − 110 °C conditions. At P20, Tskin remained lower in the − 110 °C condition (− 110 °C: 30.1 ± 0.5 °C) only, compared to pre values (Pre: 32.0 ± 0.5 °C).

Ttymp was not different between conditions at baseline, nor was between days of WBC exposure. A significant effect of time and condition (*p* < 0.05) as well as interaction between the condition of exposure and the time course of measurement (*p* < 0.05) was found. From post to P5, Ttymp in the CONT condition was higher than in the three other conditions. Ttymp remained lower (*p* < 0.05) after − 60 °C and − 110 °C exposures compared to CONT until P20 (36.4 ± 0.3; 36.4 ± 0.3 and 36.8 ± 0.2 °C, in − 60 °C, − 110 °C and CONT condition, respectively). In addition, from P5 to P20, Ttymp was systematically lower (*p* < 0.05) in − 60 °C and − 110 °C conditions compared to − 10 °C. As displayed in Fig. 2, the magnitude of the decrease in Ttymp was higher for the coldest WBC temperature.

### Thermal and comfort sensations

**Fig. 3:**
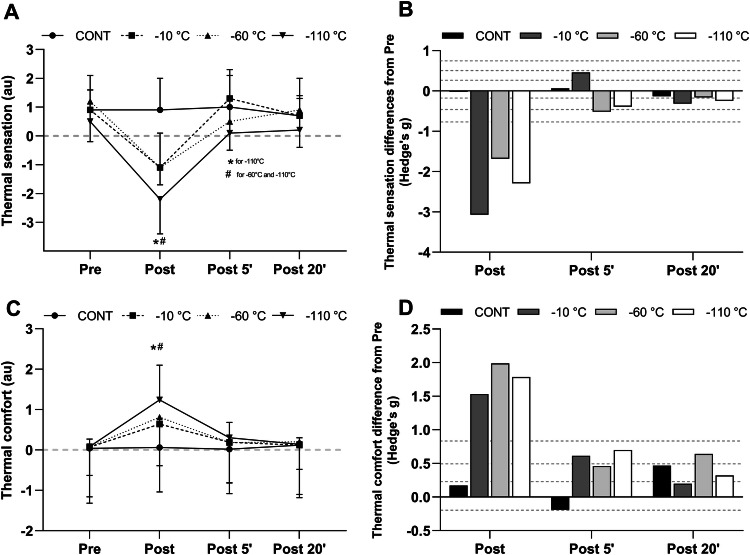
Mean and SD values of thermal (**a**) and comfort (**c**) sensation scores recorded before (pre), immediately (Post), 5 min (P5), and 20 min (P20) after a whole-body cryotherapy (WBC) session at − 10 °C, − 60 °C, − 110 °C, and in the control condition (CONT). Values are means obtained for 5 WBC sessions. *Significantly (*p *< 0.05) different from pre;# Significantly (*p *< 0.05) different from CONT. The magnitude of change (Hedge’s g) from pre for thermal (**b**) and comfort sensation (**d**). Horizontal dashed lines correspond to very small (< 0.2), small (0.2 < ES < 0.5), moderate (0.5 < ES < 0.8), large (0.8 < ES < 1.2) effects

Prior to WBC exposure, thermal sensation and comfort were identical between conditions. Since no interaction between time, condition and day of exposure was found, mean weakly values of thermal and comfort sensations are reported in Fig. 3. A main effect of time and condition as well as an interaction between time and conditions was revealed by the ANOVA. Immediately after the exposure, thermal sensation was lower in the three WBC conditions compared to pre exposure. Thermal sensation was lower (*p* < 0.001) in the − 10 °C (− 1.1 ± 0.6), − 60 °C (− 1.1 ± 1.2) and − 110 °C (− 2.2 ± 1.2) conditions compared to the CONT condition. Thermal sensation was also lower (*p* < 0.001) in the − 110 °C than in the − 10 °C condition. At P5, thermal sensation remained lower (*p* < 0.05) than in CONT in the − 110 °C WBC condition only (1.0 ± 1.1 vs. 0.1 ± 1.2 in CONT and − 110 °C condition, respectively). Thermal sensation similarly increased between post and P5 compared to post in the three WBC conditions. At P20, thermal sensations returned to pre exposure values in all conditions.

Concerning the comfort sensation, participants felt less comfortable at − 10 °C, − 60 °C and − 110 °C compared to CONT (*p* < 0.05) in post. Discomfort sensation was accentuated (*p* < 0.001) at − 110 °C (1.24 ± 0.86) compared to − 10 °C (0.64 ± 0.49) and − 60 °C (0.81 ± 0.47) condition. From P5 to P20, comfort sensation was similar between conditions.

### Blood pressure, HR and HRV indices of parasympathetic activity

**Fig. 4:**
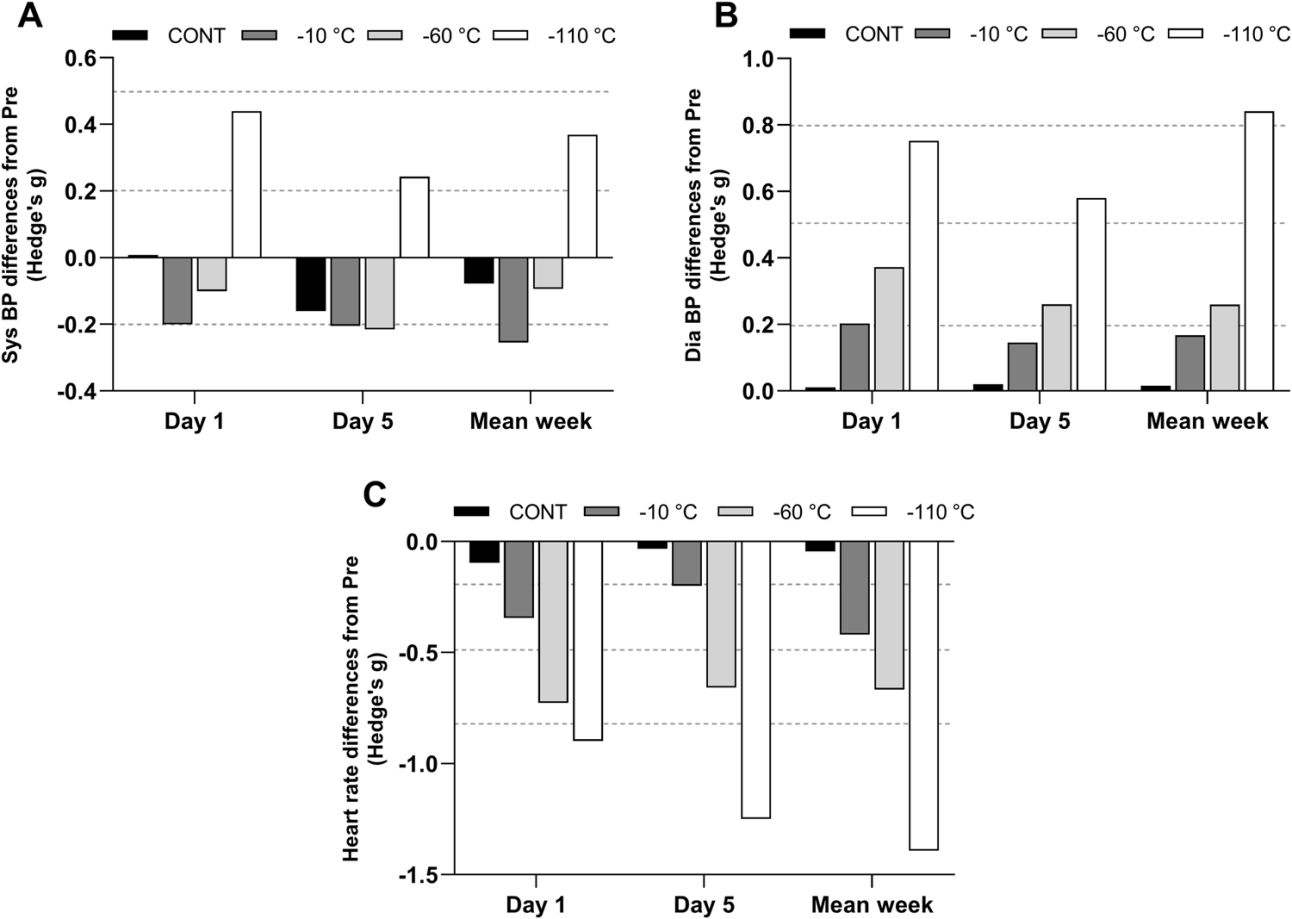
Magnitude of changes (Hedge’s g) from pre values for systolic (**a**) and diastolic (**b**) pressure and for heart rate (**c**) at day 1, day 5 and for mean weekly values. Horizontal dashed lines correspond to very small (< 0.2), small (0.2 < ES < 0.5), moderate (0.5 < ES < 0.8), large (0.8 < ES < 1.2) effects

Data for blood pressure (BP), HR and HRV indices of parasympathetic activity are reported in Table 1. The magnitude of changes for BP and HR are presented in Fig. 4. Mean values for all these variables were identical between conditions at baseline and BP, HR and HRV indices varied in the same way from day 1 to day 5. A significant interaction between time and condition was found for Sys BP and Dia BP (*p* < 0.001). Sys BP and Dia BP increased (*p* < 0.001) from pre to post exposure in the − 110 °C condition only (pre Sys BP: 123.1 ± 9.8 vs. post Sys BP: 126.8 ± 9.5 mmHg; pre Dia BP: 71.1 ± 6.9 vs. post Dia BP: 77.2 ± 7.0 mmHg).

A significant interaction between time and condition was also found for HR. When analysing mean weakly values, HR decreased from pre to post exposure in the − 10 °C (pre: 58.9 ± 10.0 vs. post: 54.7 ± 9.4 bpm, *p* < 0.001), − 60 °C (pre: 61.8 ± 12.5 vs. post: 54.0 ± 9.6 bpm; *p* = 0.01) and − 110 °C (pre: 62.4 ± 7.0 vs. post: 53.1 ± 5.7 bpm; for *P* = 0.01) conditions. In addition, in post HR was significantly lower in the − 10 °C and − 110 °C (*p* < 0.05) conditions compared to the control group.

**Fig. 5:**
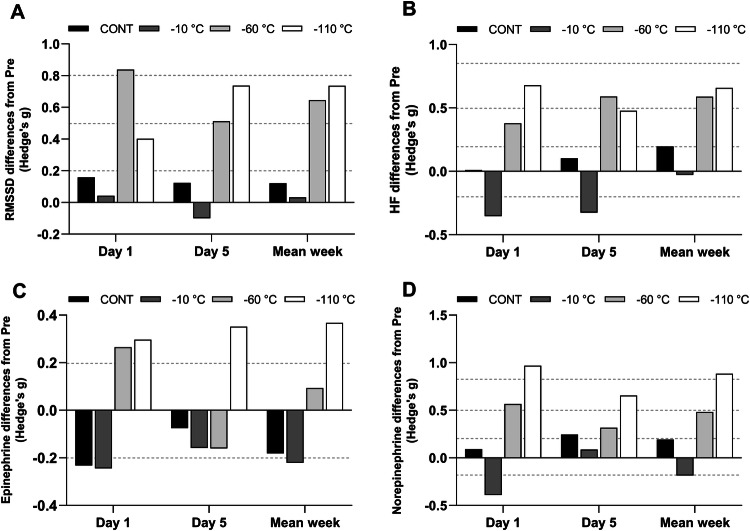
Magnitude of changes (Hedge’s g) from pre values for RMSSD (**a**), HF (**b**) epinephrine (**c**) and norepinephrine (**d**) at day 1, day 5 and for mean weekly values. Horizontal dashed lines correspond to very small (< 0.2), small (0.2 < ES < 0.5), moderate (0.5 < ES < 0.8), large (0.8 < ES < 1.2) effects

A significant effect of time and condition as well as a significant interaction between time of measurement and condition was found for the mean weakly values of RMSSD (*p* < 0.01) and for HF (*p* < 0.05). RMSSD significantly increased post exposure compared to pre exposure in the − 60 °C (pre: 40.4 ± 18.2 vs. post: 55.3 ± 25.3 ms, *p* = 0.04) and − 110 °C (pre: 48.2 ± 22.4 vs. post: 68.0 ± 28.7 ms; *p* = 0.004) conditions; whereas, HF increased post exposure only in the − 110 °C condition (pre: 606.7 ± 691.6 vs. post: 1271.1 ± 1180.5 ms^2^; *p* = 0.003). Post exposure, HF was greater (*p* < 0.05) in the − 110 °C condition (1271.1 ± 1180.5 ms^2^) compared to all others conditions (− 60 °C: 663.1 ± 546.1; − 10 °C: 314.6 ± 385.3; CONT: 583.8 ± 628.8 ms^2^). The magnitude of changes of RMSSD and HF are presented in Fig. 5a, b. Regarding the sympathovagal balance, the ANOVA also revealed an interaction between condition and time (*p* < 0.05). The post hoc analysis revealed only a decrease of the LF/HF index after the exposition at − 60 °C and − 110 °C.

### Plasma concentrations in catecholamines

Plasma concentrations in catecholamines are displayed in Table 2 and magnitudes of changes are presented in Fig. 5c, d. Norepinephrine concentration significantly increased post exposure in day 1 in the − 110 °C condition only (pre: 172.8 ± 97.5 vs. post: 352.0 ± 230.7 ng L^−1^; *p* < 0.05; whereas, it was a trend in day 5 (pre: 160.9 ± 120.2 vs. post: 293.2 ± 245.2 ng L^−1^; *p* = 0.15). This increase was confirmed when averaging values collected in days 1 and 5 (pre: 166.8 ± 86.3 vs. post: 322.6 ± 222.3 ng L^−1^; *p* < 0.001). Catecholamine concentration did not change significantly at any time point in the other experimental conditions and there was no statistical difference between conditions (Table 3).

**Table 2:** Changes in cardiovascular parameters following the different whole-body cryotherapy (WBC) conditions and compared with the control condition in days 1 and 5, and for the week

		Day 1				Day 5				Mean over week			
		CONT	− 10 °C	− 60 °C	− 110 °C	CONT	− 10 °C	− 60 °C	− 110 °C	CONT	− 10 °C	− 60 °C	− 110 °C
Systolic BP (mmHg)	Pre	125.5 ± 11.5	121.7 ± 5.8	119.7 ± 12.1	122.0 ± 9.7	121.7 ± 13.5	122.7 ± 10.0	117.3 ± 12.3	122.6 ± 9.9	124.1 ± 12.6	122.2 ± 7.4	119.5 ± 11.2	123.1 ± 9.8
	Post	125.6 ± 13.1	120.2 ± 8.3	118.5 ± 10.7	126.5 ± 9.9*	119.7 ± 10.2	120.9 ± 6.5	114.7 ± 10.7	125.2 ± 10.5	123.1 ± 12.0	120.8 ± 8.0	118.4 ± 10.0	126.8 ± 9.5*
Diastolic BP (mmHg)	Pre	73.0 ± 8.8	70.4 ± 7.8	72.9 ± 7.8	72.6 ± 8.1	68.8 ± 8.7	71.6 ± 8.3	70.9 ± 7.7	72.2 ± 8.9	71.3 ± 8.6	70.0 ± 7.7	71.2 ± 6.1	71.1 ± 6.9
	Post	73.1 ± 9.8	72.0 ± 7.3	76.0 ± 8.2	78.7 ± 7.5*	69.0 ± 9.9	72.8 ± 7.4	72.7 ± 5.4	77.3 ± 7.9	71.4 ± 9.0	71.2 ± 6.9	72.8 ± 5.9	77.2 ± 7.0*
Heart rate (beats min^−1^)	Pre	58.8 ± 7.9	57.4 ± 8.8	60.0 ± 14.0	60.6 ± 9.7	60.3 ± 12.1	56.9 ± 13.2	62.4 ± 14.0	60.7 ± 8.4	59.2 ± 10.0	58.9 ± 10.0	61.8 ± 12.5	62.4 ± 7.0
	Post	58.0 ± 8.1	54.2 ± 9.0*	50.9 ± 9.6*	52.4 ± 7.7*	59.9 ± 11.4	54.4 ± 10.8	53.9 ± 10.5*	51.2 ± 5.9*	58.8 ± 10.1	54.7 ± 9.4*	54.0 ± 9.6*	53.1 ± 5.7*
RMSSD (ms)	Pre	40.5 ± 30.9	57.9 ± 50.3	40.9 ± 20.1	55.3 ± 43.2	44.3 ± 36.9	54.8 ± 44.7	37.2 ± 21.3	47.9 ± 25.9	45.1 ± 35.2	58.6 ± 39.5	40.4 ± 18.2	48.2 ± 22.4
	Post	49.8 ± 34.1	60.8 ± 75.2	60.1 ± 23.5	73.0 ± 40.9	51.2 ± 38.0	50.3 ± 41.0	55.1 ± 41.9	72.6 ± 37.1	51.5 ± 39.1	59.9 ± 39.7	55.3 ± 25.3*	68.0 ± 28.7*
HF (ms^2^)	Pre	272.9 ± 312.4	490.5 ± 622.4	453.0 ± 477.4	822.5 ± 1098.6	605.1 ± 753.4	498.1 ± 866.0	306.7 ± 282.2	656.5 ± 695.1	466.3 ± 509.3	326.5 ± 439.2	398.5 ± 268.3	606.7 ± 691.6
	Post	275.8 ± 227.4	290.7 ± 445.8	598.3 ± 203.8	2280.9 ± 2692.3	690.0 ± 821.5	268.5 ± 401.4	694.9 ± 839.4	1201.2 ± 1377.8	583.8 ± 628.8	314.6 ± 385.3	663.1 ± 546.1	1271.1 ± 1180.5*
LF/HF	Pre	1.8 ± 2.3	3.5 ± 3.8	2.3 ± 1.5	2.7 ± 2.4	1.9 ± 1.3	3.0 ± 3.1	2.4 ± 3.1	2.5 ± 2.9	2.1 ± 1.5	2.8 ± 2.9	2.3 ± 1.6	2.5 ± 1.7
	Post	2.1 ± 2.4	2.1 ± 1.0	1.5 ± 1.0	1.6 ± 1.7	1.4 ± 1.3	2.6 ± 2.0	1.4 ± 1.0	1.4 ± 1.5	2.2 ± 1.9	2.2 ± 2.1	1.3 ± 1.0*	1.5 ± 1.7*

Data are presented as mean ± SD

*Systolic BP* Systolic blood pressure, *Diastolic BP* Diastolic blood pressure, *RMSSD* root-mean square difference of successive normal R–R intervals, *HF* High Frequencies power density

*Significantly (*p *< 0.05) different from pre condition

**Table 3:** Changes in plasmatic catecholamine concentrations after the different whole-body cryotherapy (WBC) conditions and compared with the control condition in days 1 and 5

		Day 1				Day 5				Mean (1st and 5th days)			
		CONT	− 10 °C	− 60 °C	− 110 °C	CONT	− 10 °C	− 60 °C	− 110 °C	CONT	− 10 °C	− 60 °C	− 110 °C
Epinephrine (ng L^−1^)	Pre	58.3 ± 46.3	120.6 ± 75.8	80.6 ± 67.9	40.1 ± 12.9	48.0 ± 55.7	91.4 ± 55.1	79.7 ± 51.7	32.5 ± 27.9	53.2 ± 45.0	106.0 ± 61.5	80.2 ± 54.8	36.3 ± 17.4
	Post	46.5 ± 50.8	103.8 ± 54.2	99.5 ± 68.5	46.9 ± 28.1	44.6 ± 28.4	82.8 ± 48.0	71.6 ± 45.0	43.0 ± 29.3	45.6 ± 34.7	93.3 ± 47.2	85.5 ± 55.3	44.9 ± 26.7
Norepinephrine (ng L^−1^)	Pre	259.0 ± 94.5	370.5 ± 194.6	238.3 ± 82.3	172.8 ± 97.5	273.3 ± 165.8	312.4 ± 146.3	233.8 ± 80.8	160.9 ± 120.2	266.1 ± 124.8	341.5 ± 152.1	236.1 ± 71.0	166.8 ± 86.3
	Post	270.6 ± 145.0	300.6 ± 143.4	287.7 ± 84.9	352.0 ± 230.7*	314.8 ± 157.8	326.1 ± 150.7	267.8 ± 121.1	293.2 ± 245.2	292.7 ± 139.1	313.4 ± 138.5	277.8 ± 92.9	322.6 ± 222.3*

Data are presented as mean ± SD

*Significantly (*p* < 0.05) different from pre condition

## Discussion

Using a four-group parallel design, we investigated the acute (one session in one day) and repeated (one session per day over five consecutive days) effects of three different WBC conditions (− 110 °C, − 60 °C, − 10 °C, ambient temperature) on the ANS response. The main finding was that only the most extreme WBC condition (− 110 °C) induced an increase in parasympathetic activity. This was observed through a systematic decrease in HR following each exposition, an increase in the RMSSD HRV indicator over the week and an increase in norepinephrine blood concentration on day 1. RMSSD also increased over the week in the − 60 °C condition, whilst the increase in norepinephrine concentration recorded for the − 110 °C condition was no longer significant in day 5. These results are in line with the hypothesis of Louis et al. (2015) which suggested that the magnitude of the ANS stimulation could be dependent on the cold intensity. Another point to consider is that the autonomic response to cold was lowered on day 5 as indicated by the absence of blood catecholamines rise after the fifth WBC session.

To the best of our knowledge, this study is one of the first to investigate the effect of three different WBC temperatures on skin and tympanic temperature and the subsequent modulation of the ANS. As expected, the greatest decrease in skin temperature was recorded in the − 110 °C condition and was still significant 20 min after exposure. Mean skin temperature decreased by 42%, 27% and 12% in the − 110 °C, − 60 °C and − 10 °C condition, respectively. These results are consistent with the previous studies examining the effect of WBC on skin temperature. Cuttell et al. (2017) showed a 34% decrease in mean skin temperature after 2-min whole-body exposure at − 110 °C. Similarly, Hausswirth et al. (2013) recorded a 43% reduction in skin temperature following three-minute whole-body exposure at − 110 °C. In addition, Louis et al. (2015) found a decreased mean skin temperature of 26% after 3-min whole-body exposure at − 60 °C. As mentioned previously, a reduction in skin temperature is the trigger for a cascade of physiological reactions leading eventually to an increased parasympathetic tone activation. The stimulation of cold-sensitive cutaneous thermoreceptors results in a sympathetic stimulation mediated by the release of norepinephrine. Peripheral vasoconstriction is, thus, initiated, leading to a redirection of the blood volume toward the core organs, which is responsible for an increase in blood pressure. Compensatory mechanisms downregulating the blood pressure are then activated, leading to a vagally mediated bradycardia (Leppäluoto et al. 2008).

In this study, the systolic and diastolic blood pressure significantly rose post exposure in the − 110 °C condition only. The diastolic blood pressure was significantly higher post exposure in the − 110 °C compared to the other conditions. The magnitude of changes in blood pressure was similar throughout the 5 days. Similar results were found by Hausswirth et al. (2013) after a − 110 °C WBC session. In addition, Louis et al. (2015), found a significant increase in diastolic blood pressure but not in systolic blood pressure after a − 60 °C WBC session. Westerlund et al. (2004a, b) previously studied the impact of three WBC temperatures (− 10 °C, − 60 °C and − 110 °C) on blood pressure. In contrast to our results, these authors found a significant increase in blood pressure at − 10 °C with a stronger effect with the lowest temperature. This difference with our study may be explained by the fact that in the present study, blood pressure was measured 8 min after the exposure; whereas, it was measured immediately post exposure in Westerlund et al. (2004a, b). Fonda et al. (2014) also tested four different durations of WBC (1 min 30 s, 2 min, 2 min 30 s, 3 min) at temperatures ranging from − 130 to − 170 °C. Blood pressure increased in similar proportion between WBC conditions whatever the duration of exposure was, suggesting that the temperature of exposure might have more effect on blood pressure than the duration of exposure.

In this study, the ANS responses to WBC were evaluated using HRV indices of parasympathetic activity (HF and RMSSD); while, sympathetic activity was evaluated through blood catecholamine concentrations (epinephrine and norepinephrine). RMSSD was significantly higher post exposure compared to pre exposure in both − 60 °C and − 110 °C conditions and HF significantly increased post exposure only for the − 110 °C condition. These observations suggest a greater stimulation of the parasympathetic branch with the lowest temperature of exposure. In comparison, Hausswirth et al. (2013) examined the effect of PBC and WBC at − 110 °C on the HRV and blood catecholamine response. These authors found a larger decrease in mean skin temperature after WBC and associated it with a stronger stimulation of the ANS, especially of the parasympathetic tone. Several studies also demonstrated that acute exposure to WBC at temperatures ranging from − 60 to − 120 °C significantly increased the parasympathetic activity (Westerlund et al. 2006; Zalewski et al. 2014; Douzi et al. 2018). In the present study, the effects of WBC on HRV indices of parasympathetic activity were similar after one and five exposures. Previous research investigating the effect of repeated exposure to WBC on the HRV also suggested that the cold-induced parasympathetic stimulation would not diminish nor increase after 5 daily exposures (Louis et al. 2015). In a cold acclimation protocol (daily exposition to cold air for 10 days), Mäkinen et al. (2008) also reported no difference in HRV responses between the first and the last days.

Regarding the evolution of blood catecholamines concentration in response to cold exposure, we found no effect of WBC on the epinephrine concentration; while a significant increase in norepinephrine concentration was measured after the first WBC session at − 110 °C. After the fifth exposure, no significant differences were found for plasma norepinephrine concentration between pre and post exposure in all conditions even though a trend to an increase was observed in the − 110 °C condition. Similar findings were described by Hausswirth et al. (2013) who noticed in their study a significant rise in norepinephrine following a WBC session but no increased epinephrine concentration. Louis et al. (2015) later confirmed these results and also found a lower increase in norepinephrine concentration after 5 daily exposures compared to the increase following the first exposure. Also, in contrast to the results of the present study, the increase in norepinephrine release occurred in the − 60 °C condition. However, the increase was moderate compared to studies using lower temperatures as reported by Louis et al. (2015) therefore suggesting that the adrenergic response is associated with the intensity of the cold stimulus. The blood catecholamines response was also previously studied in the context of cold-air habituation. Following acute exposure to cold air and, thus, before habituation, a significant rise in norepinephrine is generally recorded. Such increase is then reduced following several exposures (Leppäluoto et al. 2001, p. 200; Mäkinen et al. 2008). As the cold-induced increase in plasma norepinephrine concentration is the result of the sympathetic stimulation, the diminished norepinephrine responses observed after repeated exposure indicates a reduction of the sympathetic activation. The stimulation of the sympathetic nervous system induces the release of norepinephrine which is involved in the cold-induced vasoconstriction through the stimulation of α-adrenergic receptors of the vascular smooth muscle cells. Leppäluoto et al. (2008) proposed that the rise of circulating norepinephrine may be linked to a suppression of the pain signal at the spinal level. Hence, we can hypothesise that the release of norepinephrine could be responsible for the analgesic effect of WBC, which is classically reported in the literature.

## Conclusion

The use of WBC is spreading in multiple contexts. Whether it is to sooth joint pain, aid post-exercise recovery or facilitate sleep, the principle of WBC is to induce a sudden decrease in skin temperature which will be the trigger for changes of the ANS activity. The magnitude of these changes in response to different WBC modalities remained to be examined. Hence, we proposed to evaluate the effects of different temperatures of WBC applied acutely (1 session) or chronically (5 sessions in 5 consecutive days) on the modulation of the ANS. Our main findings suggest that one WBC session at -110 °C is required to stimulate the autonomic nervous system as inferred through an increase in plasma catecholamine concentration. The concomitant increase in HRV indices also suggests the parasympathetic tone became predominant after one WBC session at − 110 °C. After the 5th session, the increase in plasma catecholamine concentration was not significant and the HRV response was lowered, suggesting a habituation effect. When the data were averaged over 5 days, a predominance of the parasympathetic tone was observed following WBC sessions at − 110 °C. As such, WBC would be particularly recommended in the form of short protocols (1 week) such as during tapering weeks prior to sporting competitions when a rapid and acute increase in parasympathetic activity is sought.

## Abbreviations

ANOVA: Analysis of variance
ANS: Autonomic nervous system
BP: Blood pressure
CONT: Control condition
Dia BP: Diastolic blood pressure
EDTA: Ethylene diamine tetraacetic acid
ELISA: Enzyme-linked immunosorbent assay
ES: Effect size
HF: Power density of high frequency
HR: Heart rate
HRV: Heart rate variability
LF/HF: Low-frequency/high-frequency spectral bands ratio
PBC: Partial-body cryotherapy
RMSSD: Root-mean square difference of successive normal R–R intervals
R–R: R–R heart rate intervals
SD: Standard deviation
Sys BP: Systolic blood pressure
Tskin: Skin temperature
Ttymp: Tympanic temperature
WBC: Whole-body cryotherapy
